# Molecular Identification and Disease Management of Date Palm Sudden Decline Syndrome in the United Arab Emirates

**DOI:** 10.3390/ijms20040923

**Published:** 2019-02-20

**Authors:** Khawla J. Alwahshi, Esam Eldin Saeed, Arjun Sham, Aisha A. Alblooshi, Marwa M. Alblooshi, Khaled A. El-Tarabily, Synan F. AbuQamar

**Affiliations:** 1 Department of Biology, United Arab Emirates University, Al Ain 15551, UAE; 200413061@uaeu.ac.ae (K.J.A.); arjunsham@uaeu.ac.ae (A.S.); 201110117@uaeu.ac.ae (A.A.A.); 201416798@uaeu.ac.ae (M.M.A.); 2 Khalifa Center for Genetic Engineering and Biotechnology, United Arab Emirates University, Al Ain 15551, UAE; esameldin_saeed@uaeu.ac.ae; 3 School of Veterinary and Life Sciences, Murdoch University, Murdoch, Western Australia 6150, Australia

**Keywords:** date palm, disease management, *Fusarium solani*, pathogenicity, sudden decline syndrome, UAE

## Abstract

Date palm orchards suffer from serious diseases, including sudden decline syndrome (SDS). External symptoms were characterized by whitening on one side of the rachis, progressing from the base to the apex of the leaf until the whole leaf dies; while the internal disease symptoms included reddish roots and highly colored vascular bundles causing wilting and death of the tree. Although three *Fusarium* spp. (*F. oxysporum*, *F. proliferatum* and *F. solani*) were isolated from diseased root samples, the fungal pathogen *F. solani* was associated with SDS on date palm in the United Arab Emirates (UAE). *Fusarium* spp. were identified based on their cultural and morphological characteristics. The internal transcribed spacer regions and large subunit of the ribosomal RNA (ITS/LSU rRNA) gene complex of the pathogens was further sequenced. Pathogenicity assays and disease severity indices confirm the main causal agent of SDS on date palm in the UAE is *F. solani*. Application of Cidely^®^ Top (difenoconazole and cyflufenamid) significantly inhibited the fungal mycelial growth in vitro and reduced SDS development on date palm seedlings pre-inoculated with *F. solani* under greenhouse conditions. This is the first report confirming that the chemical fungicide Cidely^®^ Top is strongly effective against SDS on date palm.

## 1. Introduction

Date palm (*Phoenix dactylifera* L.) is an economically important crop that is mainly cultivated in tropical and subtropical regions. Date palm fruits possess high nutritional and therapeutic values with significant antioxidant, antiproliferative, and antimicrobial properties [1]. Traditionally, date palm is cultivated in the Arabian Peninsula including the United Arab Emirates (UAE), which is now one of the top 10 producers of date palm worldwide [2]. The UAE produced 825,300 and 671,891 tons of dates in 2010 and 2016, respectively, which represents a decrease of 18% in the production levels. This can be attributed to increasing production costs, unfavorable climatic changes, and disease pressures. Despite the decline in production, the increasing demand for date palm still outweighs the annual yield, making the UAE a leading producing and exporting country of dates. Therefore, it is vital to address the ongoing threats to date palm production in the UAE and around the world.

Fungi are known as the most causal agents of diseases on date palm trees [3]. Black scorch and sudden decline syndrome (SDS; also known as Fusarium wilt) are among the devastating diseases of date palm. In the UAE, the soil-borne fungal pathogen *Thielaviopsis punctulata* was found few years back to cause black scorch disease on date palm [4]. On the other hand, the causal agent of SDS remained uncertain for many years due to different *Fusarium* spp. associated with it, or mixing the symptoms with other wilt diseases. For example, 10 fungal species were associated with date palm roots that may infect date palm trees individually or in combinations to cause diseases in the UAE [5]. Although SDS has been reported to negatively affect date palm plantations, the causal agent of this disease is still ambiguous and disputed amongst researchers in the UAE and elsewhere. *Fusarium* classification and nomenclature system has gone through its unsteadily transformation; and consequently, researchers have not been able to come to a consensus on which *Fusarium* spp. is the appropriate forma specialis (f. sp.) for SDS pathogen. There are several studies that suggest *Fusarium oxysporum*, *F. moniliforme*, *F. proliferatum*, and *F. solani* to be associated with SDS in different parts of the world [6,7,8,9,10,11]. The vascular fusariosis, commonly known as Bayoud, caused by *F. oxysporum* f. sp. *albedinis* is the most destructive fungal disease of date palm in North Africa particularly Algeria, Morocco and Mauritania [12,13]. Bayoud, however, has never been reported in the UAE or any country of the Arabian Peninsula.

Despite having different *Fusarium* spp. linked with disease symptoms, SDS causes the most damage in date palm plants that live in warm, dry regions [14]. *Fusarium* spp. are soil-borne vascular wilt pathogens, and disease cycles of most Fusarium wilts can be divided into dormant, parasitic and saprophytic stages [15]. The dormant stage comprises inhibition and germination of resting structures in soil. The parasitic stage comprises penetration of roots, colonization of the root cortex and endodermis, movement to the xylem; colonization of the xylem tissues of stems and leaves, symptom expression and, finally, death of the host. The saprophytic stage is the formation of resting structures in the dead host [14]. Plants growing in cooler, wetter areas still show symptoms but in slower rates. This disease is contagious and may infect other trees nearby. In general, the fungus causing SDS in plants can persist in soil for several years without a susceptible host. Spores often enter plants through roots and are transported to the rest of plant tissues through the vascular system [9]. The pathogen attacks the xylem, reducing water uptake and the tree gradually shows signs of water scarcity. The pathogen can also spread through mechanical means e.g., pruning tools, or contaminated seedlings from unsanitary nurseries [15,16]. In general, SDS can be recognizable by similar symptoms on most cultivars of date palm [17]. Due to limitation in water transport in vascular tissues, fronds, or leaves of the tree are the first to display signs of infection. The opportunistic fungus usually attacks old, weak, or injured plants. Depending on the severity of the disease, initial symptoms occur on the lowest outer leaves of the middle crown, and discoloration of leaves from pale green to yellow usually starts at the base of the palm fronds and moves upwards [18]. When disease progresses, leaves turn white on one side before developing on the other side of the leaf. This is associated with crinkling at the ends of leaflets, and finally leaves dry and eventually die. The dying process may take a few days to several weeks. Similar symptoms will re-appear on other younger leaves. Once the symptoms of decline are clearly seen, it is difficult to reverse the progress of the disease. So far, SDS has no cure for date palm. 

On the other hand, careful management programs and good horticultural practices may reduce the pathogen impacts and extend the life of the tree [19]. Such practices can include pruning the infected parts and burning the symptomatic fronds. Since many fungal pathogens invade host tissues through mechanical injury or natural openings, wounds should be avoided. Regular maintenance and proper fertilization are also recommended to increase the health and vigor of date palm trees [20]. Yet, the use of chemicals continues to be the major strategy to mitigate the menace of crop diseases, regardless of its ecological problems and public health concerns. Foliar applications with benzimidazole- or copper-based fungicides can reduce SDS incidence at early stages of infection [21,22]. Except for a few cases, biological control agents (BCAs) such as *Trichoderma* spp. is not sufficiently effective against *Fusarium* spp. in date palm [21]. Therefore, minimum chemical fungicide application can be used. Thus, integrated disease management (IDM) programs can be implemented to manage SDS which combine cultural, chemical and biological control with resistant cultivars of date palm [23]. Our aim in this study was to isolate, identify the *Fusarium* spp. and evaluate the efficacy of available fungicides to protect seedlings of date palm from SDS. This research shed more light on this critical disease and how the pathogen is able to produce such devastating outcomes on date palm trees. We reported the evaluation of systemic chemical fungicide treatments against *F. solani* in the greenhouse as a reliable approach to reduce the economic losses associated with SDS. Future research on the disease agent and its ability to cause damage and spread on date trees will further assist in the development of effective IDM strategies to manage this disease. 

## 2. Results

### 2.1. Symptoms of SDS on Date Palm

**Figure 1:**
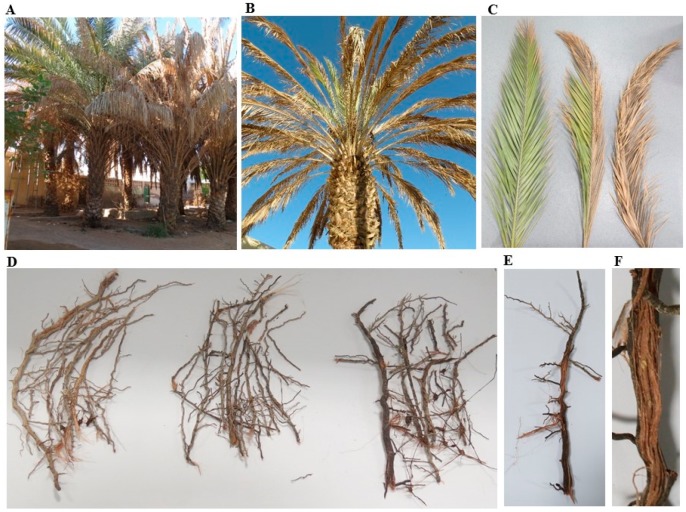
Date palm trees showing symptoms of sudden decline syndrome. Symptoms on a group of trees (**A**); and whole tree (**B**) of date palm. Progress of symptoms on fronds (**C**) and roots (**D**) of diseased plants. Brown-colored, dry roots (**E**); and redness of vascular tissues (**F**). In (**A**–**F**), naturally infested date trees with *Fusarium* spp.

Symptoms of SDS were noticed on manifested date palm trees from Al Wagan, Al Ain, UAE (Figure 1A). Although the pathogen was observed to attack different parts of the date palm trees, plant tissues were severely affected. The symptoms started with orange yellowish coloring for the rachis (frond’s midrib), followed by the leaflets (pinnae). This disease usually progresses from outer frond whorls toward central younger fronds, and from base to apex (Figure 1B). In general, drying begins from the terminal part in a single frond to cover the whole frond (Figure 1C). The affected leaves turn pale green and then to a yellowish color. The discoloration continues outward and the color becomes white on one side of the frond until the whole leaf is completely white (Figure 1C).

Under severe conditions, roots show reddish color (Figure 1D,E), fronds start drying up and the terminal bud is affected. In most cases, external symptoms are usually associated with highly colored vascular bundles (Figure 1F). Eventually, the entire tree dies within few months. Similar symptoms of decline then begin to appear on other leaves and pinnae. Thus, all palms are rarely found infected in an orchard at same time (Figure 1A). These symptoms on date palm are typical of the SDS that is known to be caused by a soil-borne fungus *Fusarium* spp.

### 2.2. Cultural and Morphological Identification of Fusaium *spp.* Associated with SDS

**Figure 2:**
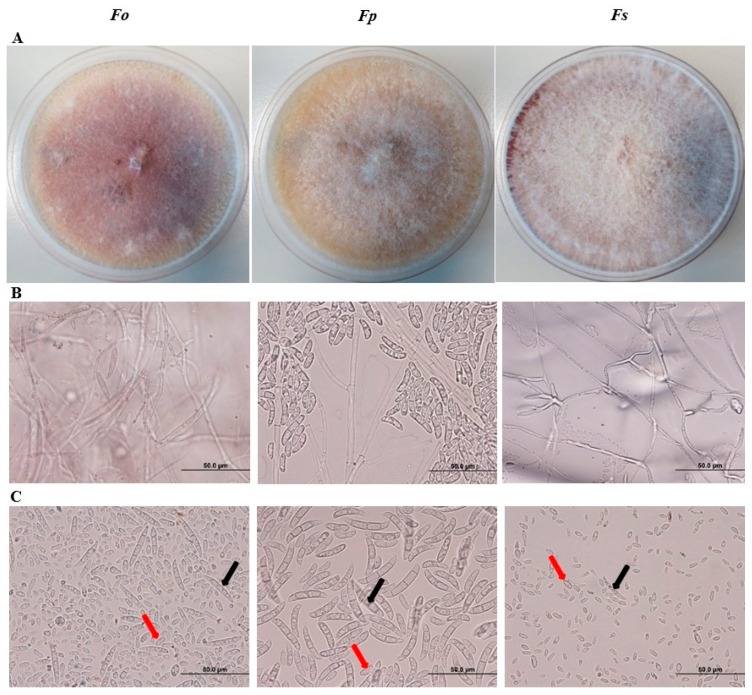
Morphological phenotypes mycelium and conidia of *Fusarium* spp. Mycelia of *Fusarium* spp. growing on a 10-day old PDA culture plate (**A**). Mycelium growth (**B**); and microconidia (red arrow) and macroconidia (black arrow) (**C**) of *Fusarium* spp. In (**A**–**C**), mycelia and conidia are from a 10-day old PDA culture. *Fo*, *F. oxysporum*; *Fp*, *F. proliferatum*; *Fs*, *F. solani*.

We microscopically examined the sporulation of the isolated pathogens from affected tissues on potato dextrose agar (PDA) plates. Based on the color of colonies and conidial morphology identified on PDA, we predicted that the culture of the identified isolates may belong to *Fusarium* spp. Colonies of the first isolate were woolly to cottony with cream to white aerial mycelium and purple pigment (Figure 2A; Figure S1). We also observed mycelial growth and production of microconidia and macroconidia. Conidiophores had simple or branched monophialides (Figure 2B). Microconidia are generally small, 1–2 celled, hyaline with oval to oblong or reniform with slightly curved shape, measuring 9.2–11.8 × 3.3–4.8 μm (Figure 2C). On the other hand, macroconidia have 3−5 septa, and are boat-shaped to oblong and 28.0–30.5 × 3.5–5.3 μm in size. Together, this suggests that this fungal isolate could be *F. oxysporum* f. sp. *cumini* (Prasad & Patel) [24].

We also isolated *F. proliferatum* (Matsushima) Nirenberg [25]. On PDA, colonies grew rapidly, aerial mycelia were white to dark vineaceous or purple (Figure 2A; Figure S1). Conidiophores were densely branched arising laterally from aerial hyphae (Figure 2B). Although chlamydospores were absent, macroconidia were distinctly abundant (Figure 2C). The 3−5 septated, nearly straight macroconidia had a size of 25.0−56.6 × 2.6−4.6 μm. Microconidia were clavate measuring 2.2−3.0 × 6.8−8.6 μm.

Colonies on PDA of the third isolate were fast-growing with white and purple, fluffy aerial mycelia with undersurface showing a dark violet color (Figure 2A; Figure S1). Hyphae were septate and hyaline; conidiophores were unbranched (Figure 2B). The produced microconidia were thin walled, hyaline, and oval to kidney-shaped, and were measured 10.8–15.4 × 2.0–4.0 µm (Figure 2C). Macroconidia had 3–5 septa with 24.6–44.2 × 3.2–4.2 μm, with stout and falcate (curved) shaping. We speculate that *F. solani* (Martius) Saccardo [26] was one of the fungal pathogens isolated from root tissues of SDS symptomatic date palm trees. Our cultural and morphological analyses suggest that *Fusarium* spp. caused SDS. Thus, molecular identification on an isolated specimen would remove any kind of controversial identification at the species level. 

### 2.3. Molecular and Phylogenetic Classification of *Fusarium spp.*

**Figure 3:**
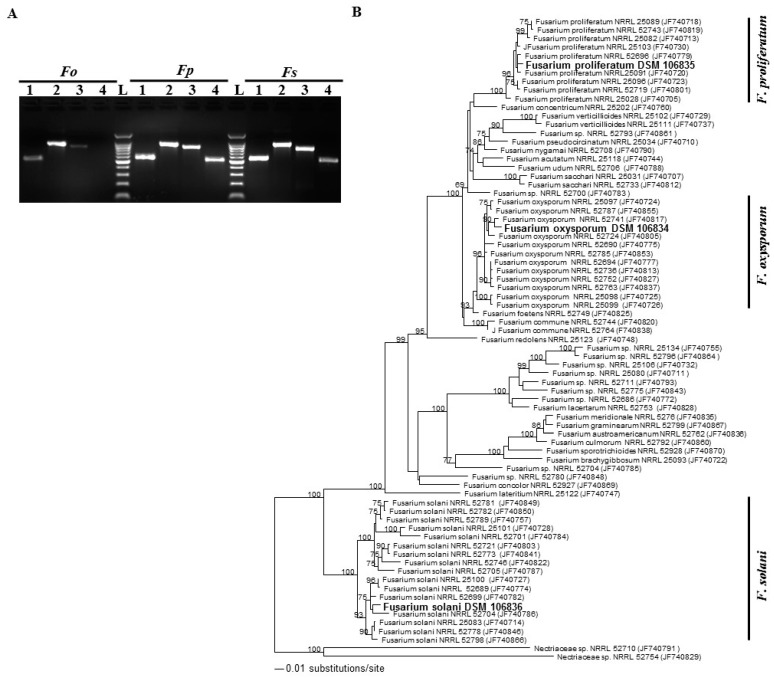
Identification of *Fusarium* spp. at the molecular level. PCR amplification of specific genomic DNA regions of infected root tissues (**A**); and dendrogram showing phylogenetic relationships among *F. oxysoprum* (*Fo*) species complex (DSM 106834), *F. proliferatum* (*Fp*) (DSM 106835), and *F. solani* (*Fs*) species complex (DSM 106836) identified in this study and other members of *Fusarium* spp. prepared by the maximum likelihood (ML) method (**B**). In (A), lanes 1–4 correspond to amplifications of *ITS*, 28S rDNA region, *TEF1-α* and *β-tubulin* in roots. In (B), the ML tree was obtained from combined *ITS/LSU* rDNA and *TEFα-1* sequence data. The specimens used in this study carry GenBank accession numbers, *Fo ITS/LSU* rDNA (MH055398), *Fo TEF1-α* (MH087478), *Fp ITS/LSU* rDNA (MH055399), *Fp TEF1-α* (MH087479), *Fs ITS/LSU* rDNA (MH055400), *Fs TEF1-α* (MH087480). Numbers at the nodes are ML bootstrap values after 100 replicates are expressed as percentages (LnL = −8908.252933). The scale bar on the rooted tree indicates a 0.01 substitution per nucleotide position. The strains from this report are indicated in bold. Nectriaceae spp. NRRL 52710 (JF740791) and NRRL 52754 (JF740829) were used as outgroups. *ITS*, *internal transcribed spacer*; 28S rDNA, large subunit (LSU) of rDNA; *TEF1-α*, *translational elongation factor 1-α*; L, DNA ladder.

We established a phylogenic analysis on the isolates obtained in this study. To identify the fungi generated from the infected tissues (Figure 2), DNA isolated from the PDA-grown mycelium of each sample was PCR amplified. Successful amplification with primer targeted to the genomic regions of *internal transcribed spacer* (*ITS*), 28S rDNA region and *translational elongation factor 1-α* (*TEF1-α*) was obtained from all putative *Fusarium* spp., and also the amplification product of *β-tubulin* gene was visible in putative *F. solani* and *F. oxysporum* specimens (Figure 3A). This confirms that *Fusarium* spp. are the pathogens frequently associated with all SDS symptoms on date palm trees. Until this report, no DNA sequences of the species collected in the UAE were available in GenBank. Therefore, the *ITS* rDNA and *TEF1-α* genes [27] were further sequenced and deposited in GenBank: *F. oxysporum ITS/LSU* rDNA (MH055398) and *TEF1-α* (MH087478), *F. proliferatum ITS/LSU* rDNA (MH055399) *TEF1-α* (MH087479), and *F. solani ITS/LSU* rDNA (MH055400) and *TEF1-α* (MH087480). We also tried to amplify the flanking DNA regions of the *Fot1* insertion sites of the pathogen *F. oxysporum* f. sp. *albedinis* from these samples. By using *pFalb11* and *pFalb28* primer pairs [28], none of the samples used in the PCR assays thus provide evidence of any diagnosis for *F. oxysporum* f. sp. *albedinis* isolates in the UAE (Figure S2).

In addition, the molecular analysis of combined sequence data of *ITS* regions and *TEF-1α* was used to determine the relationship among the obtained *Fusarium* spp. and other closely related *ITS*/*TEF1-α* sequences for the generation of the phylogenetic tree [29,30]. For the estimation of the phylogenetic tree, sequences were aligned and maximum likelihood (ML) analyses were performed. The adaptation to different plant hosts has led to the evolution of over 30 cryptic species within the *Fusarium* spp. complex [12]. The generated *ITS*/*TEF1-α* sequence belonging to the isolated strains clustered separately representing three different clades corresponding to *F. oxysporum, F. proliferatum* and *F. solani* from different sources (Figure 3B). Phylogenetic tree analysis demonstrated that three strains isolated from the UAE were placed in *F. oxysporum*, *F. Proliferatum*, and *F. solani* groups with a strong bootstrap support (100, 93, and 100%, respectively). This confirms the identity of these isolates with *Fusarium* spp. Among the studied species, our analysis revealed that our isolates *F. oxysporum* DSM 106834, *F. proliferatum* DSM 106835 and *F. solani* DSM 106836 had a sister relationship with *F. oxysporum* NRRL 52741, *F. proliferatum* NRRL 25091 and *F. solani* NRRL 52704, respectively, which distinguishes the obtained isolates *from* those belonging to other *Fusarium* or Nectriaceae spp. Our phylogenetic analysis supports that *F. oxysporum* DSM 106834, *F. proliferatum* DSM 106835 and *F. solani* DSM 106836 dominate in the UAE. Altogether, this suggests that at least one of the *Fusarium* isolates is probably the causal species of SDS on date palm.

### 2.4. Pathogenicity Tests of *Fusarium spp.* on Date Palm Seedlings

**Figure 4:**
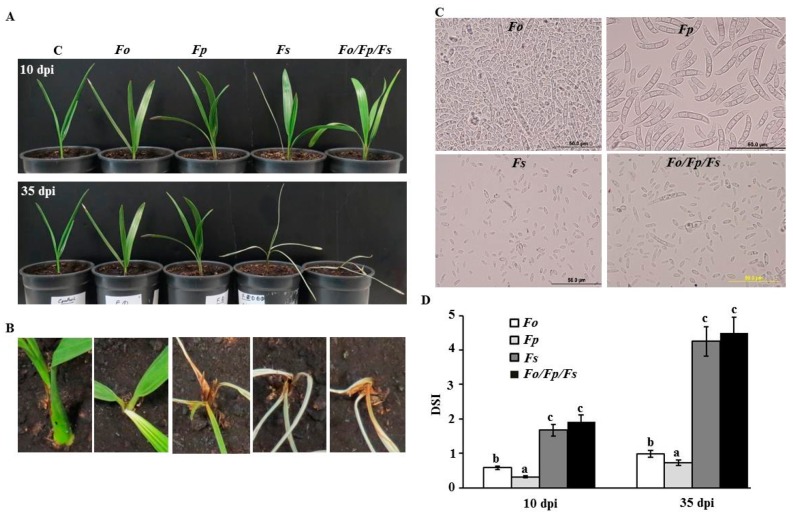
Pathogenicity and Koch’s postulate testing with *Fusarium* spp. Pathogenicity test on inoculated and non-inoculated date palm seedlings cv. Barhi at 10 dpi (top) and 35 dpi (bottom) (**A**); symptomatic tissues of the inoculated area of the seedling at 35 dpi (**B**); conidia after re-isolation of the pathogen from colonized tissues at 35 dpi (**C**); and disease severity index (DSI) of affected seedlings (*n* = 12) at 10 and 35 dpi (**D**). In (**B**), close-up views of infected stem and frond at 35 dpi of *Fusarium*-inoculated and non-inoculated seedlings. Young seedlings showing general whitening and dryness of stem and leaf tissues in *F. solani* and the three *Fusarium* spp. In (**D**), DSI is on a scale of 5: 0 = no infection, 1 = 1–10%, 2 = 11–25%, 3 = 26–50%, 4 = 51–75%, and 5 = 76–100% damage including necrosis, white area in leaves or rotting in roots. Values with different letters are significantly different from each other at *p* = 0.05. C, control (no infection); *Fo*, *F. oxysporum*; *Fp*, *F. proliferatum*; *Fs*, *F. solani*; *Fo*/*Fp*/*Fs*, all three *Fusarium* spp.; dpi, days post inoculation.

To confirm our results, pathogenicity tests using individual and combined isolated pathogens on healthy date palm seedlings (cv. Barhi) were performed and monitored for disease progress. Plants were inoculated with the conidial suspension in the root system using a 10-day old pure culture of *F. oxysporum*, *F. proliferatum*, or *F. solani* grown in PDA, while control seedlings were inoculated with sterile distilled water. We also treated the roots with the three combined conidial suspensions to determine whether SDS symptoms on date palm seedlings are similar to or stronger than in inoculations of individual *Fusarium* spp. under greenhouse conditions. Following inoculation with *F. oxysporum* or *F. proliferatum*, seedlings did not develop SDS symptoms on stem and leaf tissues (Figure 4A). However, typical symptoms of SDS such as white color and dryness of the stem and leaves appeared at 10 days post inoculation (dpi) with *F. solani*. Moreover, the disease progressed rapidly along the stem and leaves in the following days. At 35 dpi, symptoms often expressed as complete dryness of leaves and characterized by tissue necrosis in *F. solani*-inoculated plants (Figure 4A,B). Minor effect on seedlings inoculated with any of the other two *Fusarium* spp. was observed after 35 days. Similar to *F. solani*-inoculation, symptoms developed quickly and seedlings showed complete discoloration and necrosis of tissues by the end of the experiment when the three species of *Fusarium* were combined (Figure 4A,B). Control leaf tissues remained symptomless. To fulfill Koch’s postulates, the pathogens were consistently re-isolated from the disease affected tissues; thus, detected symptoms were associated with the inoculation with the pathogen *F. solani* (Figure 4C).

Based on visual observations (Figure 4A), we speculated that the three combined *Fusraium* spp. might have a synergetic effect on date palm seedlings. Disease severity index (DSI) on date palm seedlings inoculated with the three *Fusarium* spp. was not significantly different from those inoculated with *F. solani* throughout the greenhouse experiment (Figure 4D). However, pathogenic ability of *F. oxysporum* or *F. proliferatum* had less effect on the seedlings. This is evident from DSI which predicted reduced scores of affected seedlings of date palm with *F. oxysporum* or *F. proliferatum* when compared with that of *F. solani* or the combinations of *Fusarium* spp. at 10 and 35 dpi. Our data suggest that *F. solani* causes the disease on different tested tissues of date palm.

### 2.5. In Vitro Evaluation of Selected Fungicides against *Fusarium spp.*

**Figure 5:**
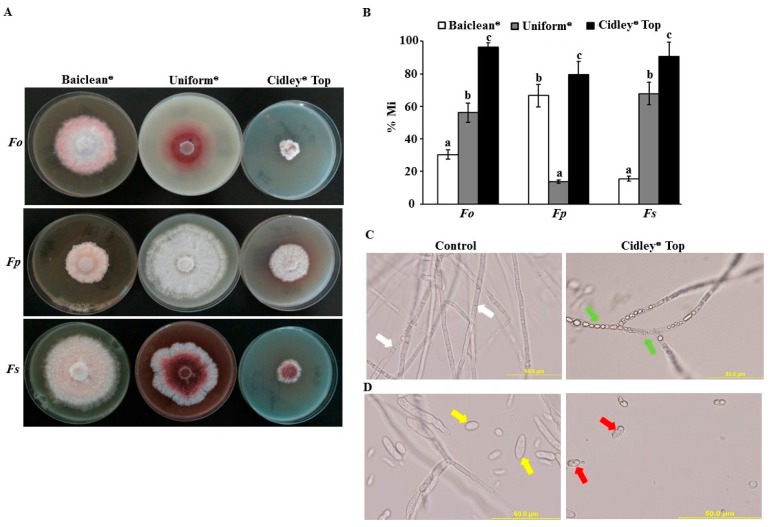
Efficacy of fungicides against *Fusarium* spp. in vitro. Effect of fungicides (250 ppm) on in vitro mycelial growth (**A**); growth inhibition rate (% Mi) of *Fusarium* spp. using 250 ppm of the fungicides after 10 days (**B**). Abnormalities in hyphal morphology, septum formation and cytoplasmic contents (**C**); and deformation of conidia (**D**) of *F. solani* following Cidely^®^ Top treatment compared to control. In (B)**,** values with different letters are significantly different from each other at *p* = 0.05; In (C)**,** white arrows indicate normal septate hyphal growth; and green arrows indicate formation of non-septate hyphal formation and cytoplasmic coagulation. In (D)**,** yellow arrows indicate normal formation of conidia; and red arrows indicate deformation of conidia. *Fo*, *F. oxysporum*; *Fp*, *F. proliferatum*; *Fs*, *F. solani*.

We evaluated the effect of Baiclean^®^ (Oligosaccharin), Uniform^®^ (Azoxystrobin and Metalaxyl-M) and Cidely^®^ Top (Difenoconazole and Cyflufenamid) fungicides on the mycelial growth of the three *Fusarium* spp. in vitro. In PDA plates, a final concentration of 0, 25, 75, 125, 250, 500, and 1000 ppm of the selected fungicides were applied (Figure S3). Interestingly, mycelial growth increased in all isolates at higher concentrations of the fungicide Baiclean^®^ (>250 ppm) compared with lower concentrations (<250 ppm). When different concentrations of Uniform^®^ fungicide were applied, we noticed similar or minimal effect on the mycelial growth of the three fungal strains (Figure S3). On the other hand, Cidely^®^ Top showed the highest mycelial growth inhibition on *Fusarium* spp. at all tested concentrations in vitro. Accordingly, the concentration of 250 ppm in the selected fungicides was further used and was considered as the most efficient concentration in the three fungicides (Figure 5A).

At 250 ppm, three fungicides were statistically evaluated for their effectiveness to inhibit the growth of three potential isolates of *Fusarium* spp. of proven pathogenicity to date palm in vitro. After 10 days of inoculation, mycelial growth inhibition rate (% Mi) of *F. oxysporum, F. proliferatum*, and *F. solani* were significantly different among the three tested fungicides at 250 ppm (Figure 5B). Thus, Cidely^®^ Top fungicide increased fungal mycelial growth inhibition at 250 ppm and showed the highest zone of inhibition, ranging between 79.5–96.3% (Figure 5A,B). This suggests that the systemic fungicide, Cidely^®^ Top, inhibited the mycelial growth of *Fusarium* spp. and that 250 ppm is the recommended dosage of this fungicide to further applied in the greenhouse experiment.

Due to the pathogenicity of *F. solani* on date palm under greenhouse conditions (Figure 4) and the effectiveness of 250 ppm of Cidely^®^ Top in vitro (Figure 5), a microscopic examination was performed to find out the mode of action of this fungicide in inhibiting the growth of this pathogen. Our results revealed that Cidely^®^ Top caused significant morphological alternations in *F. solani*. When Cidely^®^ Top was applied, we observed unusual morphological abnormalities in cultures of *F. solani* in comparison to control (no fungicide) treatment. The fungicide not only caused septal malformations and cytoplasmic coagulation in the hyphal cells (Figure 5C), but also conidial deformation in *F. solani* (Figure 5D). Altogether, Cidely^®^ Top inhibited the mycelial growth and induced morphological abnormalities of *F. solani* DSM 106836. There are several cases of chemical control of plant pathogens successfully reported under laboratory conditions, but when repeated at a greenhouse-scale, they fail to have the predicted impact [31]. 

### 2.6. Effect of Cidely^®^ Top on Date Palm Seedlings Infected with *F. solani*

**Figure 6:**
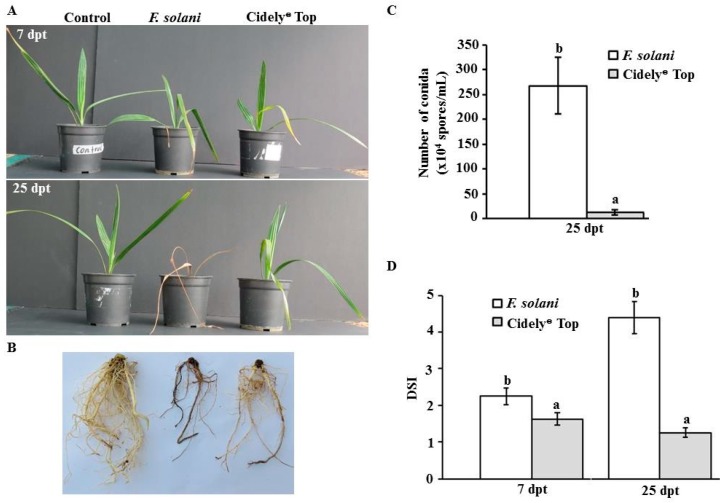
Effect of fungicide treatments on artificially inoculated date palm seedlings with *F. solani* in the greenhouse. Fungicidal suppression of Fusarium wilt disease on date palm seedlings cv. Barhi using Cidely^®^ Top fungicide at 7 (top panel) and 25 (bottom panel) dpt (**A**); recovery of root tissues previously infected with *F. solani* using 250 ppm of Cidely^®^ Top fungicide after 25 dpt (**B**). Number of conidia (×10^4^ spores/mL) (**C**) and disease severity index (DSI) (**D**) after the application of Cidely^®^ Top on date palm seedlings infected with *F. solani* (*n* = 12). In (**D**), DSI is on a scale of 5: 0 = no infection, 1 = 1–10%, 2 = 11–25%, 3 = 26–50%, 4 = 51–75%, and 5 = 76–100% damage including necrosis, white area in leaves or rotting in roots. In (C–D) Values with different letters are significantly different from each other at *p* = 0.05. In (A–D), seedlings inoculated with *F. solani* at 10 days before the fungicide treatment. Control, non-inoculated seedling; *F. solani*, infected seedlings with *F. solani* only; Cidely^®^ Top, infected seedlings with *F. solani* and sprayed with Cidely^®^ Top; dpt, days post treatment.

In the greenhouse experiments, we measured the efficacy of the most promising fungicide, Cidely^®^ Top, at 7 and 25 days post treatment (dpt) on diseased date palm plants. We first inoculated seedlings with the fungal pathogen, *F. solani*, for 10 days until plants showed visible symptoms of SDS; followed by a treatment with Cidely^®^ Top on diseased seedlings (designated as 0 dpt). At 7 dpt with the fungicide, plants started to recover and prevented further disease progression at the end of the assessment period of 25 dpt (Figure 6A). This was in contrast to diseased plants that were sprayed with water (*F. solani*). Affected plants that were treated with Cidely^®^ Top clearly showed vegetative growth recovery (Figure 6A) and developed healthy root system (Figure 6B) at 25 dpt comparable to control plants with no prior artificial infection.

We also determined the effects of the chemical fungicide, Cidely^®^ Top, on the number of conidia progressing on diseased seedlings. As expected, a steep drop in the number of conidia was remarked in Cidely^®^ Top-treated seedlings (Figure 6C). In general, the number of conidia of *F. solani* recovered from the roots of treated-date palm plants was at least 20-fold reduced when compared with that in untreated ones. Based on the level of severity of necrosis in leaves or rotting in roots, DSI was also assessed on diseased- and recovered-seedlings (Figure 6D). It is evident from our results that seedlings treated with Cidely^®^ Top fungicide had significantly lower DSI than inoculated-seedlings but without fungicide treatment at the same period of assessment (7 and 25 dpt). Together, this suggests that *F. solani* appeared to lose some of its aggressiveness as a pathogen and the severity of SDS was gradually suppressed as a disease when Cidely^®^ Top fungicide was applied on date palm plants.

## 3. Discussion

Date palm (*Phoenix dactylifera* L.) is an important and economic fruit crop in the tropical and subtropical areas and is widely grown in the UAE. Diseases, including those caused by fungal pathogens, are among the major factors that affect marketing and hinder the yield of dates [32]. Under local conditions of the UAE, roots of date palm are liable to attack by several pathogenic soil-borne fungi that cause destructive diseases such decline, wilt, neck bending and root rot [4,5]. Recent studies have linked these diseases with *F. solani*, *Lasiodiplodia theobromae*, *L. hormozganensis*, *Thielaviopsis punctulate*, and *T. paradoxa* being the most common in the UAE [4,5,33]. In the UAE, farmers might be confused between disease symptoms of black scorch and SDS on date palm. Symptoms of black scorch disease are hard black lesions on leaves, inflorescence blight, and trunk rot [4,32]. Death of trees occurs from tips or terminal buds back inwards. Typical disease symptoms of SDS in the UAE are the orange-yellowish fronds, and the drying of leaflets. In contrast to black scorch, drying usually starts from older, lower fronds toward younger, central fronds. Regrettably, the end result of untreated palm trees is death within few months in both cases.

Although several fungi have been recorded as causal pathogens of SDS on date palm worldwide [9,21,34,35], many of these studies have reported the isolation of *Fusarium* spp. from roots, fronds, and trunks of date palm trees showing wilt and decline [21,22]. For example, *F. oxysporum*, *F. proliferatum*, and *F. solani* have been most frequently found in roots displaying decline symptoms on date palm in different regions of Iraq [8,36]. In Egypt, declined date palm trees have been associated with two *Fusarium* spp., *F. solani* and *F. monliforme* [37]. A serious disease of yellowing and death of the fronds in date palm groves in Iran has also been reported to be caused by *F. solani* [9]. Three *Fusarium* spp. have been isolated from the infected fronds and roots of date palm trees, and have been identified as *F. oxysporum*, *F. proliferatum*, and *F. solani* in Saudi Arabia, particularly in Al Qassim and Al-Medina Al-Monawara regions [6]. In the UAE, 82% of the isolated fungal pathogens from date palm roots is known to be *F. solani* [5]. All previously mentioned reports are in agreement with the findings of the current study; however, no reports have linked any *Fusarium* spp. as the causal agent of SDS on date palm in the UAE. Therefore, an investigation of the causal agents of SDS on date palm and the remedy of this disease has to be done.

Due to the drying manner of fronds that displayed similar symptoms and disease severity of Bayoud (Fusarium wilt) as in North Africa [38,39], molecular diagnostic techniques using PCR [28] can eliminate the occurrence of Bayoud disease. Here, we confirm that there is no Bayoud disease and the fungus, *F. oxysporum* f. sp. *albedinis*, does not exist in the UAE. In view of the fact that three *Fusarium* spp., *F. oxysporum* f. sp. *cumini* (Prasad & Patel), *F. proliferatum* (Matsushima) Nirenberg and *F. solani* (Martius) Saccardo [24,25,26] were isolated from root tissues of infested date palm trees showing SDS symptoms. Our cultural, morphological, and molecular phylogenetic analyses revealed that *F. oxysporum* f. sp. *cumini* DSM 106834, *F. proliferatum* DSM 106835, and *F. solani* DSM 106836 were the potential isolates associated with SDS on date palm in the UAE. Microscopic examinations of the hyphal and conidial morphology of these isolates were consistent with those previously reported [40,41,42]. In this study, individual sequences generated from the three isolates were compared with those maintained in the National Center for Biotechnology information (NCBI; www.ncbi.nlm.nih.gov) and relevant sequences were included in the subsequent phylogenetic inference. Phylogenetic congruencies of the single gene set *ITS/TEF1-α*, clustered *Fusarium* isolates in three different clades corresponding to *F. oxysporum*, *F. proliferatum*, and *F. solani*. These isolates were further selected for pathogenicity assays. 

Although there are few reports about the antagonistic effects of multiple *Fusarium* spp. on plants [43]; many others have shown synergistic effects [44,45]. The four *Fusarium* spp., *F. graminearum, F. culmorum*, *F. poae*, and *F. sporotrichioides*, causing foot and crown rot diseases on wheat (Triticum aestivum L.) have been reported to be more virulence when together [45]. Given the evidence for an arsenal of virulence factors of Fusarium spp., we hypothesized that interactions of the three species would lead to additive effects in pathogenicity on date palm. It is likely that synergism among multiple pathogens leads to more severity in disease symptoms than an individual pathogen [44]. Pathogenicity assays and DSI scores on healthy date palm seedlings inoculated with either *F. oxysporum* or *F. proliferatum* in the greenhouse did not provide evidence that these isolates cause SDS. This does not rule out the possibilities that these isolates may lead to minor disease symptoms, symptoms appearing at later stages of infection, or causing diseases under certain environmental conditions. Weather conditions are important parameters determining the production of mycotoxins such as deoxynivalenol by *Fusarium* spp. to cause diseases in plants [46]. After analyzing the morphological phenotypes amongst the isolates, no significant difference was found in the DSI between seedlings inoculated with *F. solani* vs. the three combined *Fusarium* spp. This suggests that *F. solani* is most probably the Fusarium species that plays a major role in establishing the SDS in date palm plantation in the UAE.

Because immediate and appropriate management for this destructive disease was highly required, we aimed to search for a cost-effective solution for the potential risk of SDS in the UAE. For that purpose, this research was further extended to evaluate systemic and non-systemic fungicides to potentially control the pathogen in vitro as well as in the greenhouse. In our efforts to find a successful fungicide to inhibit *F. solani*, we first tested the efficacy of three fungicides, Baiclean^®^, Uniform^®^ and Cidely^®^ Top under in vitro conditions. Although all tested chemical treatments inhibited mycelial growth of *F. solani* in the controlled laboratory experiments, the systemic fungicide, Cidely^®^ Top (difenoconazole and cyflufenamid), was the most effective chemical at a concentration of 250 ppm (Figure 5). Similarly, Cidely^®^ Top has also proven to be effective against a number of pathogens such *T. punctulata* and *L. theobromae* [33,47]. Other systemic fungicides, such as Bavistin D.F. (Carbendazim), have been reported to suppress *F. solani* and *F. mangiferae* [10,48,49].

Despite there is a body of evidences that the non-selective use of chemicals poses a perceived risk to human health and adverse effects to the environment [50], chemicals are still widespread to limit the damage on crops [51]. This is because of the ease of use, effectiveness, fast activity, and relatively low cost. Searching for eco-friendly strategies is crucial to control diseases in crops and reduce growth of pathogens. For example, IDM combines two or more antagonistic strategies of horticultural practices, fungicides, BCA and natural compounds [47,52,53]. To date, research is limited on the effectiveness of fungicide treatment to manage SDS because of the unpredictable nature of the disease [54]. Performances of the same treatments under similar laboratory–greenhouse–field studies are rare in plant science.

Our data demonstrated that Cidely^®^ Top inhibited *F. solani* at 250 ppm by affecting hyphal development, septum formation and cytoplasmic integrity. In addition, conidial formation of the fungal pathogen was also altered. In general, Cidely^®^ Top was highly effective in reducing the pathogenic activities of *F. solani*. Similar to the *in vitro* observations, Cidely^®^ Top showed a significant reduction in disease symptoms in relation to the conidia counts in Cidely^®^ Top-treated seedlings at 25 dpt in the greenhouse trials (Figure 6). This indicates that Cidely^®^ Top can be considered as a candidate fungicide for the management of *F. solani*-affected date palm trees. The result obtained for Cidely^®^ Top seems to be in agreement with previous findings that this fungicide inhibits the growth of plant pathogens [33,47]. Cidely^®^ Top, on the other hand, does not have a retarded effect on *F. magniferae* [48]. This could be attributed to many factors such as fungicide application methods, growth conditions and the nature of fungal species. The fungicide Cidely^®^ Top contains the active substance, cyflufenamid, which is known to act in the developmental stages of mycelia and spores, which may possibly contribute to the increased extent of inhibition of *F. solani*. To the best of our knowledge, this is the first study relating the assessment of Cidely^®^ Top on date palm trees infected with *F. solani*. Farmers in the UAE, the region and other date production areas suffering from this devastating disease will directly benefit from this study. Thus, the same fungicide was fairly known to be successful on other pathogenic fungi including *T. punctulata* on date palm and *L. theobromae* on mango [33,47]. Therefore, a future field experiment has to be deployed to test the efficacy of Cidely^®^ Top in ‘real’ infected date palm orchards.

In this regard, we identified *F. solani* as the main causal pathogen of SDS on date palm in the UAE. We concluded that Cidely^®^ Top was a favorable chemical means to inhibit the growth of the pathogen on date palm. Future directions for seeking environmentally-friendly applications i.e., IDM to manage SDS in date palm is on our priorities. Our long term goal is to achieve the protection of date palm health and its productivity and improve environment sustainability. Conventional breeding, plant biotechnology, and modern molecular tools [53] could also generate breakthroughs for the production of new resistant varieties to SDS.

## 4. Materials and Methods 

### 4.1. Isolation, Purification, and Culture Maintenance

*Fusarium* spp. isolated from the roots of surface sterilized symptomatic date palm trees obtained from Al Wagan area in Al Ain city (Abu Dhabi Emirate; Latitude/Longitude: 24.19/55.76) were studied in this research. No specific permissions were required for the location. Sections were made of the diseased tree roots and the pathogens were isolated. The isolated fungi were maintained on PDA (Lab M Limited, Lancashire, UK) plates supplemented with 25 mg/L penicillin-streptomycin (Sigma-Aldrich Chemie GmbH, Taufkirchen, Germany) and under growth conditions at pH 6.0 and temperature 25 °C. After seven days of incubation, the mycelia growing from the plated tissues were sub-cultured on fresh PDA and lastly purified by using hyphal-tip isolation technique [55]. Later, sub-culturing on fresh slants was done at biweekly intervals to maintain the stock cultures and preserved at 25 °C. Mycelia and conidia were observed using Nikon-Eclipse 50i light microscope (Nikon Instruments Inc., NY, USA) to characterize different fungal structures. A culture of the identified *Fusarium* spp.: *F. oxysporum* f. sp. *cumini* (Prasad & Patel), *F. proliferatum* (Matsushima) Nirenberg and *F. solani* (Martius) Saccardo [24,25,26], has been deposited in Leibniz-Institute DSMZ-Deutsche Sammlung von Mikroorganismen und Zellkulturen GmbH (Braunschweig, Germany) under the collection numbers DSM 106834, DSM 106835, and DSM 106836, respectively.

### 4.2. DNA Isolation, PCR, and Sequencing

DNA of the pathogen isolated from infected root tissues was extracted from mycelium cultured for 14 days on PDA plates at 25 °C. DNA extractions were performed using the plant/fungi DNA isolation kit (Norgen Biotek Corp., Thorold, Canada). PCR reactions contained reaction buffer, 2.2 mM MgCl_2_, 200 µM of each dNTP, 2.5 unit of Taq DNA polymerase, a 30-ng DNA template and 50 pmol of each primer, making to a final volume of 50 µL. PCR was set for 32 cycles; each cycle consisted of: 94 °C for 1 min; 58 °C for 1 min; 72 °C for 1 min. For the three *Fusarium* spp., PCR amplified target regions of *ITS* using ITS1 and ITS4 primers [56], *28S rDNA* using LRoR and LR5 [57], *TEF1-α* using EF1-728F and EF1-986R [58], and *β-tubulin* using Bt1a and Bt1b [59]. In addition, *pFalb11* and *pFalb28* belonging to *F. oxysporum* f. sp. *albedinis* were also amplified. All primer sequence sets can be found in Table S1.

### 4.3. Phylogenetic Analysis

For the analysis of the phylogenetic placement of the fungal isolate the sequences of *ITS*/*LSU* rDNA and *TEF1-α* genes were used as single gene set and concatenated two-gene set, *ITS*/*TEF1-α*. The obtained *ITS/LSU* (accession numbers MH055398, MH055399, and MH055400) *TEF1-α* (accession numbers MH087478, MH087479, and MH087480) sequences were deposited in GenBank for *F. oxysporum*, *F. proliferatum*, and *F. solani*, respectively, and were further combined for constructing the phylogenetic tree against other *Fusarium* spp. database managed by NCBI. 

The *ITS*/*TEF1-a* sequences were aligned with sequences retrieved from GenBank, representing isolates that belong to about 30 species of the genus *Fusarium* [12,33]. All sequences were compared and aligned and ML analyses were performed for estimation of the phylogenetic tree [60]. Phylogenetic trees were constructed and validated with a statistical support of the branches with 100 bootstrap resamples. The isolates used in the dendrogram were: *F. proliferatum*, *F. conentricum*, *F. verticillioides*, *F. pseudocircinatum*, *F. nygamai*, *F. acutatum*, *F. udum*, *F. sacchari*, *F. oxysporum*, *F. foetens*, *F. commune*, *F. redolens*, *F. lacertarum, F. meridionale*, *F. graminearum*, *F. austroamericanum*, *F. culmorum, F. sporotrichioides*, *F. brachygibbosum*, *F. concolor*, *F. lateritium*, *F. solani*, other *Fusarium* spp. and Nectriaceae spp.

### 4.4. Disease Assays and Pathogenicity Tests

Fungal isolates grown in PDA plates as described above were prepared for conidial suspension for inoculation purposes. The inoculums were prepared by adding 10 mL of sterile distilled water to the 14-day-old fungal culture and scrapped by sterilized scraper. The harvested conidial solution was poured into a 50 mL Falcon tube after filtering the solution twice through sterile Miracloth. The conidial concentration was determined using hemocytometer (Agar Scientific Limited, UK) and adjusted to 1 × 10^6^ conidia/mL according to [61].

Pathogenicity tests were conducted using Koch’s postulates to confirm the *Fusarium* spp. as the causal agent of SDS of date palm orchard. Six-month-old date palm seedlings (cv. Barhi), obtained from the Date Palm Development Research Unit/UAEU (DPDRU) and showing no disease symptoms, were used. The *Fusarium* spp. used were the representative of isolates of *F. oxysporum* DSM 106834, *F. proliferatum* DSM 106835, and *F. solani* DSM 106836, which were successfully isolated and identified from SDS of date palm orchard. Seedlings (*n* = 12) were inoculated with 10 mL of inoculum on roots that were separately dipped for one hour in the inoculum suspension of each *Fusarium* spp. or altogether. The control plants were dipped in 10 mL of sterile distilled water. The inoculated seedlings were covered with transparent polyethylene sheet for 72 h. Seedlings in 1.5-L plastic pots containing sterilized soil mixture (3:2:1 *v*/*v* ratio of top soil–peatmoss–sand) were grown randomly on the bench in a greenhouse with temperature of 25 °C and 60% RH, and watered two times weekly. Disease severity index on inoculated seedlings was recorded for SDS symptoms at 10 and 35 dpi using a scale of 0–5: 0 = no apparent symptoms, 1 = 1–10% necrotic or white area in leaves or rotting in roots, 2 = 11–25%, 3 = 26–50%, 4 = 51–75%, and 5 = 76–100% [62]. All experiments were independently repeated three times with similar results.

To satisfy Koch’s postulates, pieces of inoculated root tissues were removed from seedlings showing diseases symptoms at 35 dpi, surface sterilized with 70% ethanol and plated on PDA. Plates were incubated at 25 ± 2 °C and the subsequent growth was recorded. 

### 4.5. Evaluation of Fungicides against *F. solani*

The fungicide experiment was carried out as previously described [4,33,47]. These fungicides selected were Baiclean^®^ (Oligosaccharin; Baico, Beijing Multigrass Formulation Co., China), Uniform^®^ (Azoxystrobin and Metalaxyl-M; Syngenta, USA) and Cidely^®^ Top 125/15 DC (Difenoconazole and Cyflufenamid; Syngenta). Each of the obtained fungicide was dissolved in water to a final concentration of 0, 25, 75, 125, 250, 500, and 1000 ppm, and then introduced into sterilized PDA at 25 °C. To inhibit the bacterial growth, penicillin-streptomycin antibiotics was also introduced. The solution was swirled to attain homogenization status. The mixtures were dispensed aseptically into sterile Petri dishes. A sterile cork-borer (8-mm in diameter) was used to introduce the tested pathogen onto the medium without fungicide (control) or with fungicide (treatment). Cultures were incubated at 25 °C for 10 days and radial growth measurements were recorded daily. The percentage of the mycelial growth was measured and growth inhibition was calculated according to the equation

% Mi = (Mc − Mt)/Mc × 100%(1)

where; Mi = Inhibition of the mycelial growth; Mc = colony diameter (in mm) of control set; and Mt = colony diameter (in mm) of the target fungus on the medium with fungicide.

An in vivo evaluation of Cidely^®^ Top, which is the most effective fungicide, was also carried out on six-month-old date palm seedlings (cv. Barhi) grown in 1.5-L plastic pots containing sterilized soil mixture under greenhouse conditions, as described above. Seedlings were previously inoculated with 10 mL of inoculum on roots that were separately dipped for one hour in the inoculum suspension of *F. solani*, and were further kept in the greenhouse at 25 °C for 10 dpi (until disease symptoms were evident). Similar to inoculated seedlings, control plants were dipped in 10 mL of sterile distilled water. *F. solani*-inoculated plants were then either sprayed with 250 ppm of Cidely^®^ Top fungicide or with sterilized distilled water (*F. solani*). To estimate the number of conidia in the greenhouse experiment, conidia counts of known weight of affected tissues was homogenized in 5 mL of water and the suspended material was assessed using haemocytometer. For all inoculated seedlings, DSI was recorded for SDS symptoms at 7 and 25 dpt (corresponding to 17 and 35 dpi with *F. solani*) using a scale of 0–5, as described above.

### 4.6. Statistical Analysis

For the in vitro evaluation of fungicides against *Fusarium* spp., eight plates for each treatment were used. For the DSI of the pathogenicity of *Fusarium* spp. and Cidely^®^ Top treatment tests against *F. solani* in the greenhouse, three replicates for each treatment were examined and recorded. Data represent the mean ± SD from a minimum of 12 plants per replicate. Analysis of Variance (ANOVA) and Duncan’s multiple range test were performed to determine the statistical significance at *p* < 0.05. All experiments were independently repeated three times with similar results. Similar results were obtained in each replicate. SAS Software version 9 was used for all statistical analyses performed [63]. 

## Supplementary Materials

Supplementary materials can be found at http://www.mdpi.com/1422-0067/20/4/923/s1.
