# Supplementary material for: Molecular Identification and Disease Management of Date Palm Sudden Decline Syndrome in the United Arab Emirates
Source: Int J Mol Sci. 2019 Feb 20;20(4):923. doi: 10.3390/ijms20040923 (PMC6412958; doi:10.3390/ijms20040923)
Supplement: Supplementary file 1 [file ijms-20-00923-s001.pdf]

Supplementary Materials: Molecular Identification and Disease Management of Date Palm Sudden Decline Syndrome in the United Arab Emirates

Khawla J. Alwahshi, Esam Eldin Saeed, Arjun Sham, Aisha A. Alblooshi, Marwa M. Alblooshi, Khaled A. El-Tarabily and Synan F. AbuQamar

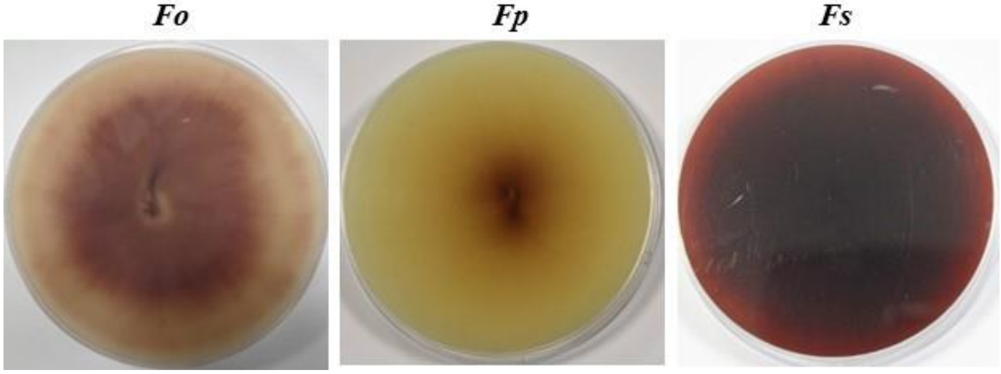

Figure S1. Sporulation of *Fusarium* spp. on a 10-day old PDA culture plate. *Fo, F. oxysporum*; *Fp, F. proliferatum*; *Fs, F. solani*.

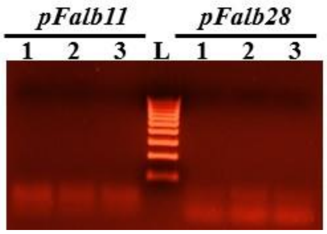

Figure S2. PCR amplification of specific genomic DNA regions of infected root tissues. Lanes 1-3 correspond to amplifications of specific primers of *F. oxysporum* f. sp. *albedenis* clone *pFalb11* and *pFalb28* in leaf tissues of seedling 1, 2 and 3. L, DNA ladder.

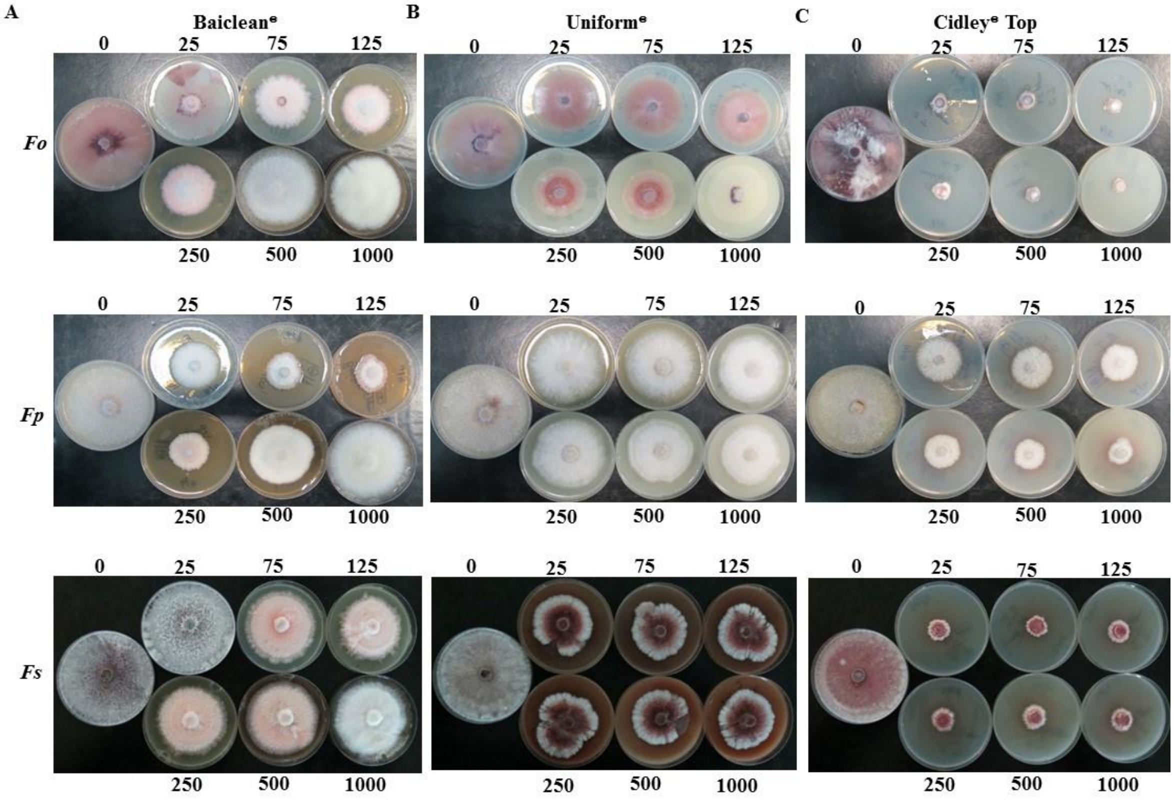

Figure S3. Growth inhibition effect of fungicides on *Fusarium* spp. Growth inhibitory effect on *Fusarium* spp. using different concentrations (in ppm) of (**A**) Baiclean®; (**B**) Uniform® ; and (**C**) Cidely® Top, on PDA pales. Data were collected 10 days after inoculation. *Fo*, *F. oxysporum*; *Fp*, *F. proliferatum*; *Fs*, *F. solani*.

Table S1. List of PCR primers (sequence 5′ to 3′) used in this study.

Description	Left primer sequence	Right primer sequence
ITS	ITS1: TCCGTAGGTGAACCTGCGG	ITS4: TCCTCCGCTTATTGATATGC
28S rDNA	LRoR: ACCCGCTGACTTAAGC	LR5: TCCTGAGGGAAACTTCG
TEF1-α	EF1-728F: TCATCGCAAGTCGAGAAGGT	EF1-986R: ACTTGAAGGAACCCTTACCG
β-tubulin	Bt1a: TTCCCCCGTCTCCACTTCTTCATG	Bt1b: GACGAGATCGTTCATGTTGAACTC
ITS/LSU	*ITS/LSU*F: TACGCCGCATCCTTGCCGAG	*ITS/LSU*R: TTCCGTAGGTGAACCTGCGG
pFalb11	FOA1: CAGTTTATTAGAAATGCCGCC	BIO3: GGCGATCTTGATTGTATTGTGGTG
pFalb28	FOA28: ATCCCCGTAAAGCCCTGAAGC	TL3: GGTCGTCCGCAGAGTATACCGGC
